# Xpert Bladder Cancer Monitor May Avoid Cystoscopies in Patients Under “Active Surveillance” for Recurrent Bladder Cancer (BIAS Project): Longitudinal Cohort Study

**DOI:** 10.3389/fonc.2022.832835

**Published:** 2022-01-27

**Authors:** Vittorio Fasulo, Marco Paciotti, Massimo Lazzeri, Roberto Contieri, Paolo Casale, Alberto Saita, Giovanni Lughezzani, Pietro Diana, Nicola Frego, Pier Paolo Avolio, Piergiuseppe Colombo, Grazia Maria Elefante, Giorgio Guazzoni, Nicolò Maria Buffi, Michael Bates, Rodolfo Hurle

**Affiliations:** ^1^ Department of Biomedical Sciences, Humanitas University, Milan, Italy; ^2^ Department of Urology, Istituto di Ricovero e Cura a Carattere Scientifico (IRCCS) Humanitas Research Hospital, Milan, Italy; ^3^ Department of Pathology, Istituto di Ricovero e Cura a Carattere Scientifico (IRCCS) Humanitas Research Hospital, Milan, Italy; ^4^ Medical and Scientific Affairs and Strategy, Oncology, Cepheid, Sunnyvale, CA, United States

**Keywords:** BIAS, active surveillance, Xpert BC, NMIBC, biomarker, cystoscopies, urine cytology

## Abstract

**Objectives:**

To test the hypothesis that patients under active surveillance (AS) for Non-muscle Invasive Bladder Cancer (NMIBC) who were negative on longitudinal re-testing by the Xpert^®^ Bladder Cancer Monitor (Xpert BC Monitor) assay may avoid unnecessary cystoscopies and urine cytology (UC).

**Subjects/Patients (or Materials) and Methods:**

This is a prospective cohort study of patients enrolled in the AS protocol for recurrent NMIBC (Bladder Cancer Italian Active Surveillance, BIAS project), whose urine samples were analyzed by Xpert BC Monitor upon entry in the study (T0). Patients who had a negative Xpert test and did not fail AS, underwent additional Xpert tests after 4 (T1), 8 (T2), and 12 (T3) months. The clinical utility of Xpert was assessed by determining the number of cystoscopies and UC that could be avoided within 1 year.

**Results:**

Overall, 139 patients were tested with Xpert at T0. Median follow-up was 23 (IQR 17–27) months. Sixty-eight (48.9%) patients failed AS, 65 (46.7%) are currently on AS, and 6 (4.3%) were lost at follow-up. At T0 57 (41.0%) patients had a negative test and 36 (63.2%) are still in AS. In patients with 2 consecutives negative Xpert tests, we could have avoided 73.9% of unnecessary cystoscopies, missing 26.4% failure, up to avoid all cystoscopies with 4 negative tests missing only 12% of failure. All the patients with negative Xpert had negative UC. Failure-free-survival at median follow-up (23 month) stratified for having 0, 1, or ≥2 negative tests was 67.0, 55.1. and 84.1, respectively.

**Conclusion:**

Our findings suggest that Xpert BC Monitor assay, when it is longitudinally repeated, could significantly reduce the number of unnecessary cystoscopies and UC during their follow-up.

## Introduction

Bladder cancer (BC) is one of the most common cancers, with non-muscle invasive bladder cancer (NMIBC) accounting for about 75% of new diagnoses (1). Progression to muscle-invasive, metastatic lethal disease occurs in approximately 15% of patients (2).

Due to the high relapse rate after treatment, patients with NMIBC typically undergo intensive follow-up based on urine cytology (UC) and in-office cystoscopy, which make BC one of the most expensive cancers per patient. In addition, UC is poorly sensitive for BC detection.

Some cancers are considered indolent, as they are either stagnant or “grow too slowly to be life threatening in even the longest of lifetimes” (2, 3). For this reason, active surveillance (AS) has been suggested as a safe “treatment” strategy for prostate and thyroid cancers that are both examples of cancers that are sometimes indolent and can be detected early through increased use of screening (4). For the same reason, the increasing detection of renal masses as an incidental finding has led to the adoption of this strategy for selected patients with small renal tumors (5, 6)

Following the same idea, in 2003 Soloway et al. suggested AS for selected recurrent LG NMIBC as a safe alternative to transurethral resection of bladder tumor (TURBT) treatment and this strategy has been successively widely adopted (7–9). Although TURBT is still the gold standard treatment, having both a diagnostic and therapeutic role, it is not devoid of complications. At the same time, AS protocols include frequent in-office cystoscopies, which are invasive procedures leading to discomfort of patients and risk of infections. For these reasons, new biomarkers for a less invasive, more sensitive, and more cost-effective diagnosis of BC are urgently needed in order to replace the low sensitivity of urinary cytology (UC) and the invasiveness of cystoscopy (10).

Xpert BC Monitor is a new mRNA-marker assay for BC follow-up after primary diagnosis and measures the levels of five target mRNAs from a cleared urine sample by real-time RT-PCR. Xpert BC Monitor automates and integrates sample processing, nucleic acid amplification, and the detection of target sequences outperforming cytology with a sensitivity and specificity of 73 and 90%, respectively (11, 12). The Xpert BC Monitor test is a CE-IVD test available in some but not all European countries and is not available in the United States.

In addition to biomarkers, imaging tools such as ultrasound and magnetic resonance imaging have been developed for the diagnosis of BC (NMIBC vs MIBC) and for recurrence (13). The use of a MRI-based standardized reporting system, the Vesical Imaging Reporting and Data System (VI-RADS) has been suggested to further improve the characterization of the tumor and peer-to-peer communication (14–17). MRI contraindications, cost, and availability is limiting the diffusion of this technique (18–21). An integration of imaging and biomarkers could improve test accuracy to select who could potentially avoid cystoscopy screening (22–24). Recently, the possibility of deferring cystoscopies and even TURBT using genetic panels has been investigated by Shkolyar et al. and Hurle et al. reported the preliminary data of the application of the Xpert BC monitor in patients enrolled in the Bladder Cancer Italian Active Surveillance (BIAS) project showing promising results (25–27).

In this study, we tested the hypothesis if a persistently negative Xpert BC Monitor test, may avoid unnecessary cystoscopies and replace UC in patients enrolled in the BIAS project for NMIBC.

## Materials and Methods

### Study Design and Population

This is a monocentric, prospective cohort study, conducted within our Institutional AS protocol for recurrent NMIBC (BIAS project-BIAS_V1.2_27.01.2018). We included patients who met the BIAS inclusion criteria and performed at least one Xpert^®^ BC Monitor test (Cepheid, Sunnyvale, CA, USA) (8). The BIAS inclusion criteria are recurrence of 1–5 tumors, tumors size <1 cm, absence of gross hematuria and negative UC for high-grade (HG) carcinoma). Exclusion criteria of this study were: previous history of a HG carcinoma, carcinoma *in situ* (CIS), positive UC or invalid test. All patients signed written informed consent and were able to withdraw from the protocol at any time and be offered the standard treatment.

The AS follow-up protocol consisted of UC and an in-office flexible cystoscopy every 3–4 months in the first year, and then every 6 months annually (28).

Urine samples were collected and analyzed with Xpert BC Monitor upon BIAS entry or in patients who meet the inclusion criteria during the follow-up cystoscopies. This study period was defined starting time (T0), then, if the test was negative and the patient did not fail AS, urinalyses were performed after 4 (T1), 8 (T2), and 12 (T3) months. To be tested consecutively, patients must have a negative test and still be enrolled on AS.

Cystoscopies were performed or supervised by two senior urologists and all images were stored in a digital repository archive. At each cystoscopy, new images were compared with the previous ones.

### “Xpert Bladder Monitor Test” Characteristics

Urinalysis was performed with Xpert BC Monitor at each surveillance set point if they met the criteria mentioned above. The urine biomarker tests measured the gene expression levels of five targets (ABL1, CRH, IGF2, UPK1B, and ANXA10) by RT-PCR according to the manufacturer’s protocol. Xpert BC Monitor results (called LDA—linear discriminant analysis) depend on a regression algorithm that utilizes the cycle threshold results of the five mRNA targets (11, 12). Each test cartridge contains internal controls to assess the quality of starting material and PCR reaction. The time to result is about 90 min. For the analyses the cut point of the manufacturer (positive test if LDA ≥0.5) or a stricter alternative (≥0.4) was used (26).

### Pathology Characteristics

All surveillance UC were performed according to the Paris classification system by experienced pathologists of our institution (29).

In cases of failure, patients underwent TURBT and all pathological specimens were reviewed by two experienced genitourinary pathologists according to the WHO and Classification of Tumors and staging according to the TNM (International Union against Cancer, 2009) (30).

The primary endpoint was to test clinical performance of the Xpert BC Monitor test, performed longitudinally, to predict AS failure free, according to the previously described criteria. Clinical utility was assessed by determining the number of in-office cystoscopies and UC that could be safely avoided within 1 year.

### Statistical Analysis

Categorical variables were represented as frequencies, while continuously coded as median and interquartile range (IQR). Patient groups were compared with chi-squared tests for categorical variables. Failure was defined as achieving one of the following criteria: increased number and/or size of lesions, occurrence of hematuria, positive/suspected UC, voluntary withdrawal from the protocol, any reason to undergo TURBT even if it resulted in negative histology. Death from other causes or loss to follow-up was not considered a failure. A sub-analysis was performed considering patients who voluntarily withdrew or had a negative TURBT result as non-fail to show a more accurate test performance.

The frequency of avoided cystoscopies was determined as the number of negative Xpert calls at one time point divided by the total number of patients with 2, 3, and 4 negatives tests, respectively. The frequency of missed failure was determined as number of failures associated with a negative test divided by the total number of negative tests.

Failure-free-survival (FFS) was estimated with life-table product limit estimates. Kaplan–Meier curves were used to describe failure free survival. Log-rank test was used to compare the distribution among patients grouped based on the number of negative tests. Univariate and multivariate logistic regression model (LRM) were used to test factors associated with failure. All p-values were two sided, and statistical significance was assumed at p <0.05. All analyses were performed using STATA^®^ (version Stata/IC 16.1; StataCorp LLC, TX, USA).

## Results

### Patients Baseline Characteristics

Overall, 139 patients performed Xpert test at T0, and were therefore enrolled in the current analysis. The median age was 73.0 years (IQR 66.0–78.0) and median follow-up from the enrolment in the study to last follow-up was 23 months (IQR 17–27). Overall, 135 (97.1%) were pTa and 4 (2.9%) pT1 at first diagnosis, and all were LG. Forty-eight patients (34.5%) had a history of bladder instillations. No patient underwent instillation in the 6 months prior to the test. Regarding last tumor recurrence, 49 (35.3%) patients were enrolled in the BIAS protocol at their first recurrence, 25 (18.0%) recurred within one year and 48 (34.5%) recurred after more than one year (**Table 1**).

**Table 1:** Baseline characteristics of the whole population.

		Total
		N = 139
**Age, median (IQR)**		73 (66–78)
**Gender, N (%)**	Female	27 (19.4)
	Male	112 (80.6)
**Hematuria, N (%)**	No	136 (97.8)
	Yes	3 (2.20)
**Smoking habit, N (%)**	Non-smoker	46 (33.1)
** **	Current smoker	33 (23.7)
** **	Former smoker	35 (25.2)
** **	Unknown	25 (18.0)
**Stage, N (%)**	pTa	135 (97.1)
** **	pT1	4 (2.90)
**Grade, N (%)**	Low grade	139 (100)
**Last Tumor Diagnosis before starting BIAS, N (%)**	Primary tumor	54 (39.1)
** **	Recurrence within 1 year	26 (18.8)
** **	Recurrence after 1year	58 (42.0)
**Adjuvant treatment? N (%)**	No	81 (62.8)
** **	Yes	48 (37.2)
**Type of adjuvant treatment, N (%)**	Mitomycin C	34 (72.0)
** **	BCG	7 (15.0)
** **	Gemcitabine	2 (4.00)
** **	Other/Unknown	4 (9.00)

BCG, Bacillus Calmette–Guerin; BIAS, Bladder Cancer Italian Active Surveillance; IQR, interquartile range.

### AS Outcome Characteristics

Sixty-one (43.9%) were already part of the BIAS project at study enrollment, while 78 (56.1%) were newly enrolled in the protocol at beginning of this study.

A total of 68 (48.9%) patients failed AS, while 65 (46.8%) did not fail AS and 6 (4.30%) were lost at follow-up or died from other causes. There was no difference in the failure rate between those already in BIAS or just enrolled in the AS protocol (p = 0.494). Causes of failure are summarized in **Table 2**. Of those who did not fail on AS, 55 (84.6%) had a stable lesion, 6 (9.23%) had no lesions found at cystoscopy, and 4 (6.15%) died from other causes (**Table 2**).

**Table 2:** BIAS outcome details, pathological features and Xpert test results by active surveillance outcome.

		Total	Failure	Not Failure	Other	p-value
		N = 139	N = 68	N = 65	N = 6	
**BIAS history, N (%)**	Already enrolled	61 (100)	30 (49.2)	27 (44.3)	4 (6.56)	0.494
	Newly enrolled	78 (100)	38 (48.7)	38 (48.7)	2 (2.60)	
**AS outcome details, N (%)**	No more lesion	6 (100)	n.r.	6 (100)	n.r.	<0.001
** **	On AS	55 (100)	n.r.	55 (100)	n.r.	
** **	Death for other cause	6 (100)	n.r.	4 (66.6)	2 (33.3)	
** **	Lost at follow-up	4 (100)	n.r.	n.r.	4 (100)	
** **	Increase in size	32 (100)	32 (100)	n.r.	n.r.	
** **	Increased number	14 (100)	14 (100)	n.r.	n.r.	
** **	Hematuria	6 (100)	6 (100)	n.r.	n.r.	
** **	UC: positive\suspected	5 (100)	5 (100)	n.r.	n.r.	
** **	Increased N° and size	3 (100)	3 (100)	n.r.	n.r.	
** **	Increased size and Hematuria	5 (100)	5 (100)	n.r.	n.r.	
** **	UC and Increase N°	1 (100)	1 (100)	n.r.	n.r.	
** **	Increased size, hematuria & UC	1 (100)	1 (100)	n.r.	n.r.	
** **	Voluntary withdrew	1 (100)	1 (100)	n.r.	n.r.	
**TURBT stage, N (%)**	Negative	9 (100)	9 (100)		0 (0.00)	0.89
** **	pTa	52 (100)	51 (98.0)		1 (2.00)	
** **	pT1	3 (100)	3 (100)		0 (0.00)	
**TURBT grade, N (%)**	Low grade	46 (100)	45 (97.8)		1 (2.20)	0.66
** **	High grade	9 (100)	9 (100)		0 (0.00)	
**Xpert Xpert 1st point, N (%)**	Negative	57 (100)	20 (35.1)	36 (63.2)	1 (1.70)	0.005
** **	Positive	82 (100)	48 (58.5)	29 (35.4)	5 (6.10)	
**Xpert Xpert 2nd point, N (%)**	Negative	34 (100)	9 (26.5)	25 (73.5)		0.13
** **	Positive	12 (100)	6 (50.0)	6 (50.0)		
**Xpert Xpert 3rd point, N (%)**	Negative	25 (100)	4 (16.0)	21 (84.0)		0.39
** **	Positive	4 (100)	0 (0.00)	4 (100)		
**Xpert Xpert 4th point, N (%)**	Negative	25 (100)	3 (12.0)	22 (88.0)		

AS, active surveillance; BIAS, Bladder Cancer Italian Active Surveillance; n.r., not reported; TUBRT, transurethral resection of bladder tumor; UC, urinary cytology.

Of patients who failed, 64 (94.1%) out of 68 underwent surgery in our institution. Forty-six (67.7%) were confirmed to have pTa LG, 6 (8.8%) had pTa HG, 3 (4.4%) had pT1 HG, and 9 (13.2%) patients had a negative finding (**Table 2**). No patient experienced a progression to pT2 stage BC.

### Xpert Test Characteristics

**Figure 1:**
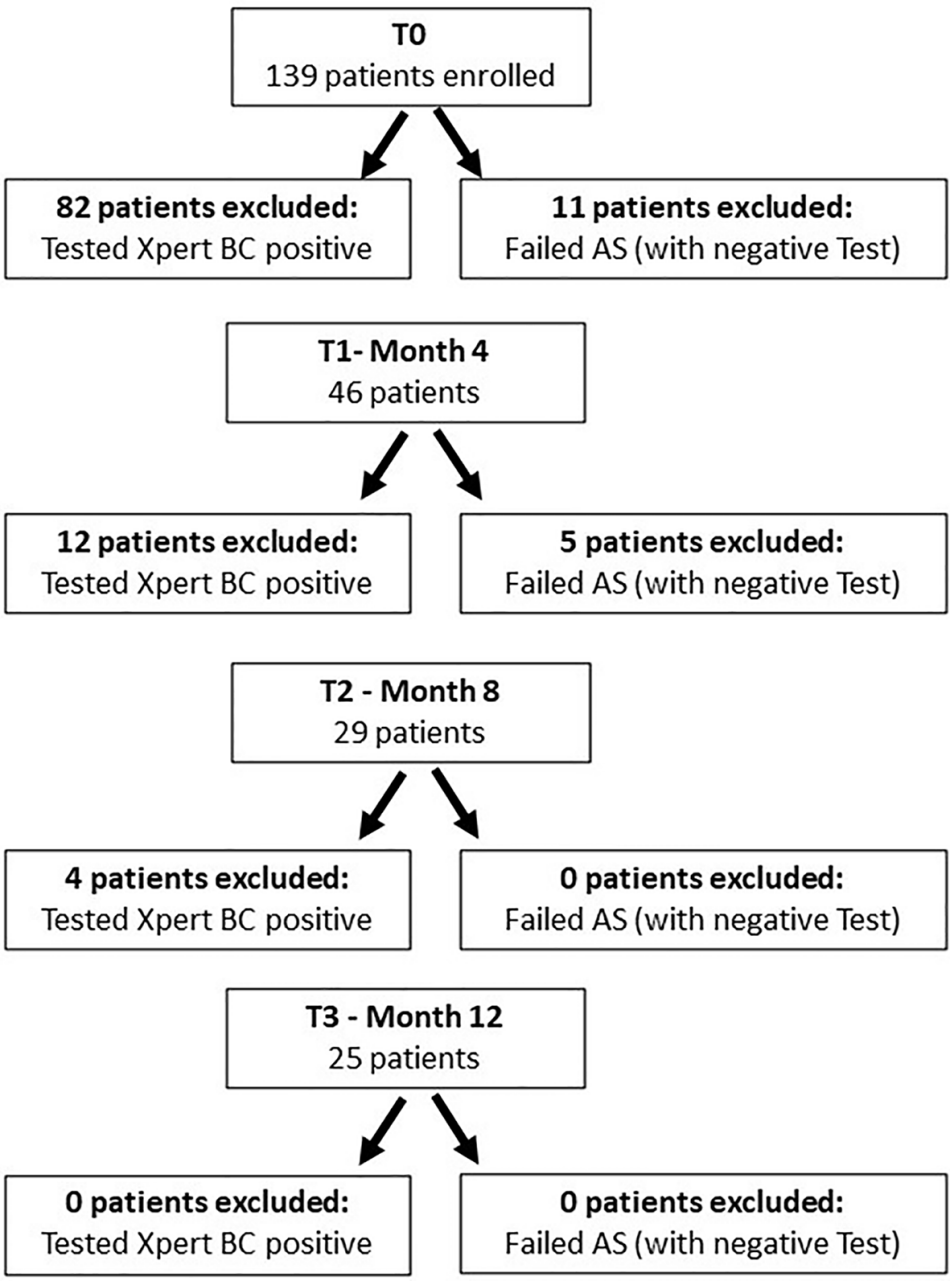
Flow chart of enrolled and who dropped out of the study. To access the next “time point”, each patient had to have tested negative on the Xpert Bladder Cancer Monitor test and had none of the active surveillance failure criteria.

At the study enrolment (T0) 57 (41.0%) patients had a negative test (manufactured LDA) and of these 36 (63.2%) are still on AS. At second surveillance (T1) 34/46 (73.9%) were still negative and of those 25 (73.5%) still on AS. At third follow up (T2) 25/29 (86.2%) remained negative and of those 21 (84.0%) still on AS. At final urine detection (T3) 25/25 were negative and 21 (84.0%) currently under AS (**Figure 1**).

### Avoiding Cystoscopies at Different LDA Cut Point and Sub-Analysis

Using the manufacturer’s LDA (>0.5), in patients with 2 consecutive negative tests, we could have avoided 73.9% of unnecessary cystoscopies, missing 26.4% of patients who failed, while after 3 negative tests can avoid 86.2% cystoscopies, missing only 16.0% of patients who failed AS. Finally, after 4 negatives tests, all of follow-up cystoscopies could have been safely avoided, missing only 12.0% of patient who failed.

Using a stricter LDA (>0.4), in patients with 2 consecutive negative tests, we could have avoided 52.2% of unnecessary cystoscopies, missing 16.7% of patients who failed, while after 3 negative tests can avoid 55.2% cystoscopies, missing only 12.5% of patients who failed AS. Finally, after 4 negative tests, 68.0% of follow-up cystoscopies could have been avoided, missing only 11.8% of patients who failed. A sub-analysis of the stratified group is shown in **Table 3**. No correlation between lesion size and LDA was found (p >0.05).

**Table 3A–B:** The data are displayed with two different LDA cut points.

(A)				
	Analysis LDA ≥0.5		Sub-analysis LDA ≥0.5	
	AVD. CYSTOSCOPIES (%)	MISSED Failure (%)	AVD. CYSTOSCOPIES (%)	MISSED “real” Failure (%)
**2 NEG tests**	73.9	26.4	73.9	23.5
**3 NEG tests**	86.2	16.0	86.2	12.0
**4 NEG tests**	100	12.0	100	8.00
**(B)**				
	**Analysis LDA ≥0.4**		**Sub-analysis LDA ≥0.4**	
	**AVD. CYSTOSCOPIES (%)**	**MISSED Failure (%)**	**AVD. CYSTOSCOPIES (%)**	**MISSED “real” Failure (%)**
**2 NEG tests**	52.2	16.7	52.2	16.6
**3 NEG tests**	55.2	12.5	55.2	6.25
**4 NEG tests**	68.0	11.8	68.0	5.89

The number of avoided cystoscopies and missed failure are referred to by two different definitions of failure. Failure according to the definition of the BIAS protocol and failure according to the histological findings; (A) LDA ≥0.5, (B) LDA ≥0.4.

All the patients with negative Xpert had negative UC, supporting the clinical utility of the test versus UC.

Considering the kinetics of LDA between time periods T0–1, T1–2, and T2–3: 69.6, 65.5, and 72.0% of patients, respectively, had a higher value.

**Figure 2:**
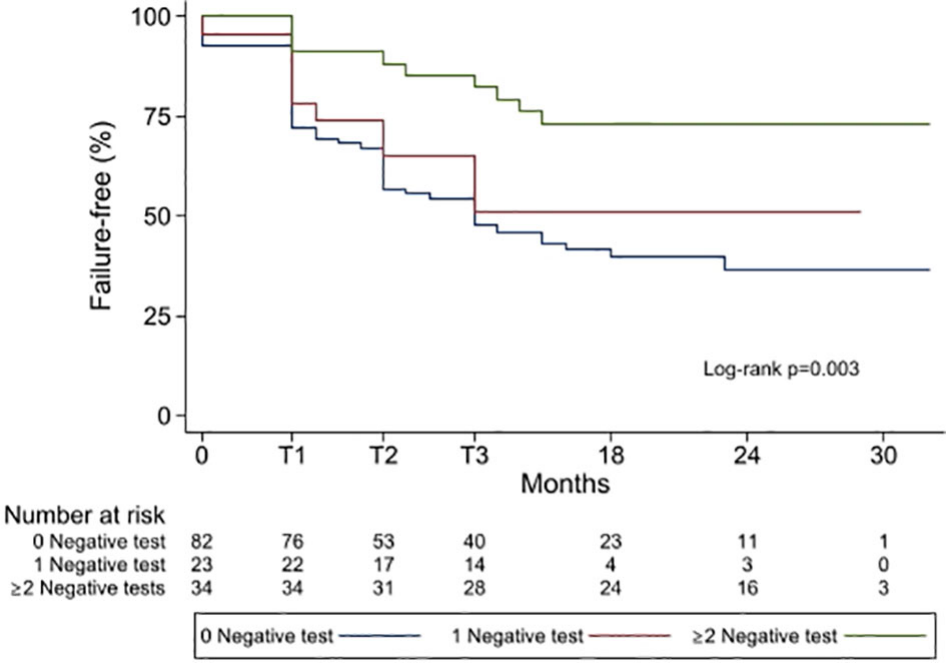
Kaplan–Meier showing failure-free frequencies by group of patient having 0 negative test, 1 negative test or ≥2 negative tests. T1 = 4 months, T2 = 8 months, T3 = 12 months.

FFS at 23 months (median FU) stratified for having 0, 1, or ≥2 negative test was 67.0% (95%CI 57.7–77.2), 55.1% (95%CI 31.2–73.7), and 84.1 (95%CI 65.9–93.1) respectively, (Log-rank p = 0.003, **Figure 2**). Median time to failure was 13 (IQR 5–23) months.

Basic characteristics such as age, biological-sex, smoking habits, and the number of negatives were tested at univariate and multivariate LRM for AS failure prediction. Having ≥2 negative Xpert tests was protective against failure and the only variable associated with failure in both models OR 0.25 (95%CI 0.105–0.614, p = 0.002), OR 0.25 (95%CI 0.103–0.643, p = 0.004), respectively.

## Discussion

In this study, the hypothesis was that having a persistently negative test could predict AS non-failure and that Xpert BC Monitor could replace UC and even avoid cystoscopy or at least extend cystoscopy follow-up interval.

In fact, our major finding is that for patients with two negative tests, cystoscopies could have been avoided in 74% of cases, missing only 26% of the tumors, and for patients with three negative tests, even up to 84% of cases, missing only 16% of cancers. Furthermore, we did not find a single patient with negative tests and positive UC.

UC is a diagnostic test whose accuracy remains limited. The introduction of the new Paris classification improved both UC sensitivity (SE), which is ranging from 34 to 95%, and negative predictive value (NPV), ranging from 46 to 86%. However, specificity (SP) remains lower than 70% (31, 32). Accuracy varies with the experience of pathologists in interpreting the findings of urinary cytology (33). For these reasons, a lot of effort has been made in developing new tools with high precision and, at the same time, a low cost, such as UC (34).

Xpert BC Monitor has an added value because it does not require an interpretation, especially in those low-volume laboratories where the experience of the cytopathologist can influence the result of UC.

As a new low-cost test, Xpert BC Monitor has been developed with the intent of increasing accuracy of BC diagnosis (35). It is proven that Xpert BC Monitor has high SE for HG NMIBC, as reported by a study by Elsawy et al., involving 181 patients. That study shows a 73.7% SE, 79.6% SP, 29.8% positive predictive value (PPV), and 96.3% NPV, demonstrating superior diagnostic performance in detecting relapses for NMIBC patients compared to UC (36). Similarly, the study of D’Elia et al. on a cohort of 230 patients demonstrates that Xpert BC Monitor SE for HG NMIBC is significantly higher than UC (57.1% vs 85.5%) (37). Additionally, a study by van Valenberg et al. shows that Xpert BC has a higher NPV for bladder cancer patients during follow-up than both UC and another molecular test, Urovysion, with a NPV of 93, 86, and 88%, respectively (38).

Thus, our study is concordant with previous literature findings and extends the results to a specific population of patients under active surveillance. In fact, we demonstrated the superiority of Xpert BC Monitor in detecting low-grade tumors over UC.

In the era of the SARS-CoV-2 pandemic, a urinary biomarker that reduces the discomfort of patients and risk becomes essential, especially for frail elderly patients with BC (39).

Furthermore, in-office flexible cystoscopy is costly, invasive, unpleasant, and carries the risk of severe urinary tract infections and urethral strictures (40, 41). It also has low SE and SP for small and/or flat lesions, such as CIS, depending on the experience of the operator (42).

Besides Xpert BC Monitor, other mRNA-based biomarkers have been validated, like Cxbladder monitor, that Koya et al. show can reduce follow-up cystoscopies by 77.8%, confirming the utility of urinary molecular biomarkers (43). In our study, by replacing the UC with Xpert BC Monitor performed over time, we could have avoided up to 73%, 86.2%, or even up to all unnecessary cystoscopies, depending on whether the patient had two, three, or four consecutive negative tests. The panorama of emerging urinary biomarkers in the diagnosis and surveillance of NMIBC is wide, but none are currently included in daily clinical practice probably due to the availability, the cost that limits their use (44–47).

In the study of Shkolyar et al., the utility of Xpert BC Monitor is evaluated for risk stratification in cystoscopy triaging for patients presenting with hematuria, allowing for early identification of patients with intermediate and high-risk BC (25). Differently, in our study, we used it in a longitudinal follow-up setting for patients with AS, demonstrating its clear superiority over UC, and to the best of our knowledge, our study has the longest AS cohort follow-up, and is the only one using the bladder-biomarker in a repeated setting (46, 48).

The use of MRI including functional sequences has been suggested to be able to discriminate between NMIBC and MIBC (14, 15, 49–51). This imaging technique could potentially result in overstaging as tumor-associated fibrosis or inflammation can mimic the low signal intensity of the muscularis propria (52). To overcome these limitations, the VI-RADS score has been developed to help defining and standardizing the grade of BC invasion (14, 15, 51). However, potential drawbacks MRI have to be considered, such as costs and causes of artifacts (53). Recent studies also showed concordance between experienced and inexperienced readers (50), and a prospective analysis has also been published confirming the previous results (51).

Additionally, we found a fairly high failure-free survival at median time of follow up for patients with 2 or more negative tests (84.1%), which further supports the real possibility of a safe referral to cystoscopy when a patient has at least two persistently negative tests.

There are some limitations to acknowledge. First, the sample size is relatively small. Second, the population is a low-risk BC sub-cohort, so the results cannot be extended to all BC patients. However, our cohort represents patients with AS quite appropriately, including both newly enrolled patients for the BIAS project and long-time enrolled patients. In addition sub-analyses at different LDAs may be biased by the fact that only patients with a negative manufacturer’s cut point have repeated the test.

## Data Availability Statement

The raw data supporting the conclusions of this article will be made available by the authors, without undue reservation upon request.

## Ethics Statement

The studies involving human participants were reviewed and approved by the Humanitas Research Hospital ethics committee: BIAS project-BIAS_V1.2_27.01.2018. The patients/participants provided their written informed consent to participate in this study.

## Author Contributions

ML and RH contributed to the conception and design of the study. VF, ML, and RH organized the database. VF, MP, PD, NF, and PA collected the data. VF and MP performed the statistical analysis. VF wrote the first draft of the manuscript. All authors contributed to the article and approved the submitted version.

## Conflict of Interest

The authors declare that the research was conducted in the absence of any commercial or financial relationships that could be construed as a potential conflict of interest.

## Publisher’s Note

All claims expressed in this article are solely those of the authors and do not necessarily represent those of their affiliated organizations, or those of the publisher, the editors and the reviewers. Any product that may be evaluated in this article, or claim that may be made by its manufacturer, is not guaranteed or endorsed by the publisher.
